# Bibliometric analysis of hot literature on neural circuit research

**DOI:** 10.1002/ibra.12144

**Published:** 2023-12-21

**Authors:** Ruo‐Lan Du, David H. Mauki, Zong‐Fu Zuo

**Affiliations:** ^1^ Department of Anatomy, Histology and Embryology Jinzhou Medical University Jinzhou Liaoning China; ^2^ National‐Local Joint Engineering Research Center of Translational Medicine, West China Hospital Sichuan University Chengdu Sichuan China; ^3^ Department of Microbiology, Parasitology and Biotechnology, College of Biomedical Sciences Sokokine University of Agriculture Morogoro Tanzania

**Keywords:** bibliometric analysis, functional connectivity, neural circuits, neurological brain diseases, publication output

## Abstract

Numerous brain diseases have been attributed to abnormalities in the connections of neural circuits. Exploration of neural circuits may give enlightenment in treating some intractable brain diseases. Here, we screened all publications on neural circuits in the Web of Science database from 2007 to 2022 and analyzed the research trends through VOSviewer, CiteSpace, Microsoft Excel 2019, and Origin. The findings revealed a consistent upward trend in research on neural circuits during this period. The United States emerged as the leading contributor, followed by China and Japan. Among the top 10 institutions with the largest number of publications, both the United States and China have a strong presence. Notably, the Chinese Academy of Sciences demonstrated the highest publication output, closely followed by Stanford University. In terms of influential authors, Karl Deisseroth stood out as one of the most prominent investigators. During this period, the majority of publications and citations on neural circuit research were found in highly influential journals including NEURON, NATURE JOURNAL OF NEUROSCIENCE, and so forth. Keyword clustering analysis highlighted the increasing focus on neural circuits and photogenetics in neuroscience research, and the reconstruction of neural circuits has emerged as a crucial research direction in brain science. In conclusion, over the past 15 years, the increasing high‐quality publications have facilitated research development of neural circuits, indicating a promising prospect for investigations on neurological and psychiatric diseases.

## Graphical abstract

This study presents a comprehensive overview and analysis of the global research trends in neural circuit research. Over the past 15 years, there has been a remarkable surge in the quantity and quality of publications within this field. The exploration of neural circuit mechanisms has shown promise in treating various nervous system diseases, thus establishing itself as a burgeoning field. This breakthrough offers novel ideas and methodologies for treatment, instilling hope in patients and holding great potential in addressing diabetes and its associated complications.

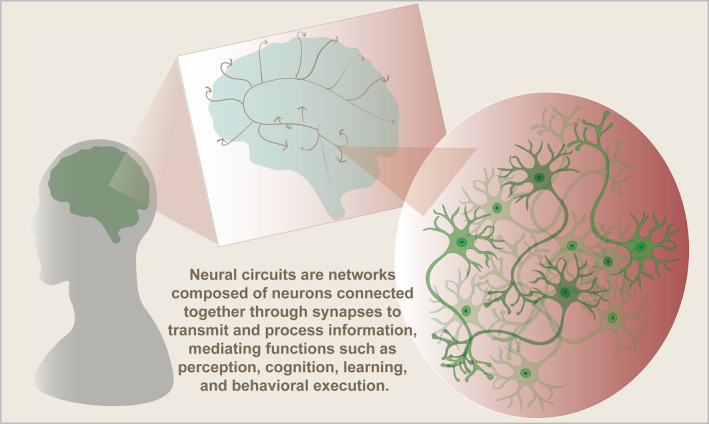

## 1 INTRODUCTION

The neural circuit is a complex network of nerve fibers that transmits, processes, and regulates brain information and establishes connections by regulating synaptic connections between neurons.^1^ The neural circuit is the basis of our perception of the world, thinking, and behavior, and it realizes brain function by transmitting brain signals to generate “behavior” mechanisms. Neural circuit reconstruction is a method to treat nervous system diseases. It can promote communication and signal conduction between neurons by activating specific neural circuits in the nervous system so as to achieve a therapeutic effect.^2^, ^3^ It has been found that the pathogenesis of many intractable neurological diseases is closely related to the abnormalities of neural circuits, such as cerebral palsy, Parkinson's disease, Alzheimer's disease (AD), and so forth.^4^, ^5^, ^6^ It is well known that disruptions or abnormalities in the corticospinal circuits can lead to motor impairments and movement disorders.^7^ There are varieties of neural circuits involved in the pathogenesis of AD; for example, the network function of the hippocampal–entorhinal circuit is closely linked with cognitive tasks.^8^ Therefore, understanding the structure and function of these circuits is essential for studying and treating various neurological conditions. The principles of promoting the recovery of central nervous system disorders mainly include functional recovery driven by plasticity, the cell signaling mechanism of axon budding, the key time of recovery after injury, and the mechanism of cell replacement strategy.^1^, ^9^, ^10^ Within the macro framework of functional circuit reconstruction, the strategic regulation of microcell behavior should provide the basis for most nerve repair strategies.^11^ In addition, in recent years, with the emergence of overall solutions for the structure and function research of neural circuits such as photogenetics, chemical genetics, electrophysiology, and virus tracing.^12^, ^13^, ^14^, ^15^ This makes cell regulation, neural circuit reconstruction, and disease behavior “visualized,” which greatly promotes the research of nervous system diseases. Based on this, scientists solved the mystery of repairing and functional remodeling of damaged neural circuits, specifically, repairing damaged neural circuits in the brain and remodeling neural functions by transplanting stem cells or neural cells derived from stem cells.

Transplanted neural cells derived from stem cells can specifically repair the damaged substantia nigra striatum circuit in the adult brain and improve the behavioral disorders of Parkinson's disease model animals.^16^ After transplanting highly specialized neural stem cells into the lesions of mice with spinal cord injuries, the neural stem cell graft can self‐assemble into a neural network similar to the spinal cord and functionally combine with the host nervous system.^17^ Transplanted neural cells derived from human embryonic stem cells can accurately reconstruct the damaged basal ganglia circuits for repairing brain structure and function and can improve the motor dysfunction of hypoxic‐ischemic encephalopathy mouse model for a long time.^11^ The basic mechanism of learning and memory deficits in AD patients involves neural circuit dysfunction. The circuit mechanism of β‐amyloid protein and tau pathology during the progression of AD is another strategy to treat the refractory neurodegenerative disease AD that leads to learning and memory deficits.^18^, ^19^, ^20^ Although most of these studies are carried out at the animal level, this suggests that it is a very active intervention direction for us to study some refractory neurological diseases based on the neural circuit mechanism.

Bibliometrics is a cross‐statistical method that integrates mathematics, statistics, and philology to predict global research trends in a certain research field.^21^, ^22^ With the great progress made in the study of the structure and function of neural circuits, the number of relevant publications has increased sharply, but relevant bibliometric research has not yet been reported. In this study, we analyzed the publications on neural circuit research in the Web of Science database from 2007 to 2022, including the statistical analysis of publications, countries, institutions, journals, authors, and keywords, aiming to analyze the heated trend of neural circuit research, draw the main functional neural circuit diagram of current research, and provide direction for future research.

## 2 METHODS

**Figure 1:**
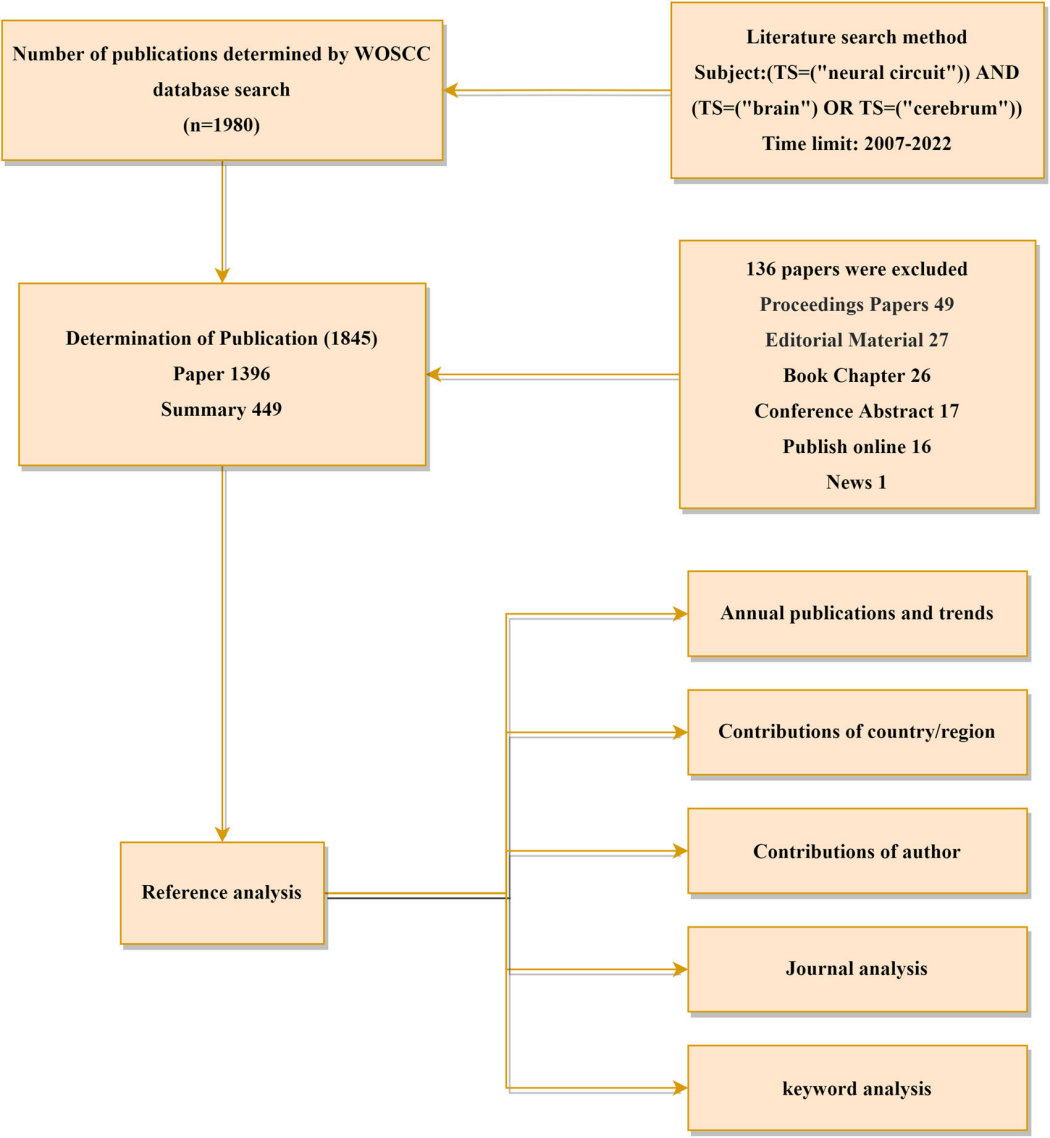
Flowchart of the screening process of neural loop research. TS, terms; WOSCC, Web of Science Core Collection. [Color figure can be viewed at wileyonlinelibrary.com]

We extracted the literature related to neural circuits from the Web of Science Core Collection (WOSCC) from 2007 to 2022, set the search term as (TS = (neural circuit) AND (TS = (brain) OR TS = (cerebrum)), and selected the literature between 2007 and 2022, excluding all types of articles except synthesis and papers. All the searched information was exported in the form of “plain text,” including countries/regions, institutions, authors, journals, and keywords (Figure 1). In addition, the journal citation report (JCR) category (2023) of each journal was searched separately. All searches were conducted on March 10, 2023. In the JCR category, we have extracted the objective and quantitative indicators of the cited times and *H* index two important information from WOS (https://webofscience.clarivate.cn/wos/alldb/basic-search) to evaluate the scientific output and academic achievements of individuals or research groups. VOSviewer 1.6.17 was employed to conduct country or regional coauthorship analysis, institutional coauthorship analysis, author coauthorship analysis, and keyword contribution analysis as previously described.^23^ In the network generated by the VOSviewer, node size represents the number of releases, and larger nodes represent more releases. Links between nodes represent the correlation between parameters (countries/regions, institutions, authors, or keywords), and the thickness of links represents their strength. In addition, we also conducted a journal coauthorship analysis through CiteSpace 6.2.1, with parameters set as follows: time‐slicing was chosen from 2007 to 2022, year per slice(1), and all options in the term source were selected; node types were selected one at a time with the selection criteria (g‐index, g2 ≤ *k* Si ≤ gci, *k* ∈ *Z* + , *k* = 25).^24^ Each node in the figure indicated an observation, including country, institution, author, co‐cited literature, and keywords. If the publication is issued in cooperation with several countries, institutions, and authors, then each country, institution, and author is taken into account, which is the basis for the network of institutional, national, and author cooperation. The importance of nodes in the network is quantitatively determined by their total link strength (TLS) with other nodes. Finally, we use Microsoft Excel 2019 and Origin for data processing and data visualization, including annual publication count, citation frequency analysis, and calculation of average citation time and *H* value for each project of each country, institution, or author.

## 3 RESULTS

### 3.1 Trends in publications

**Figure 2:**
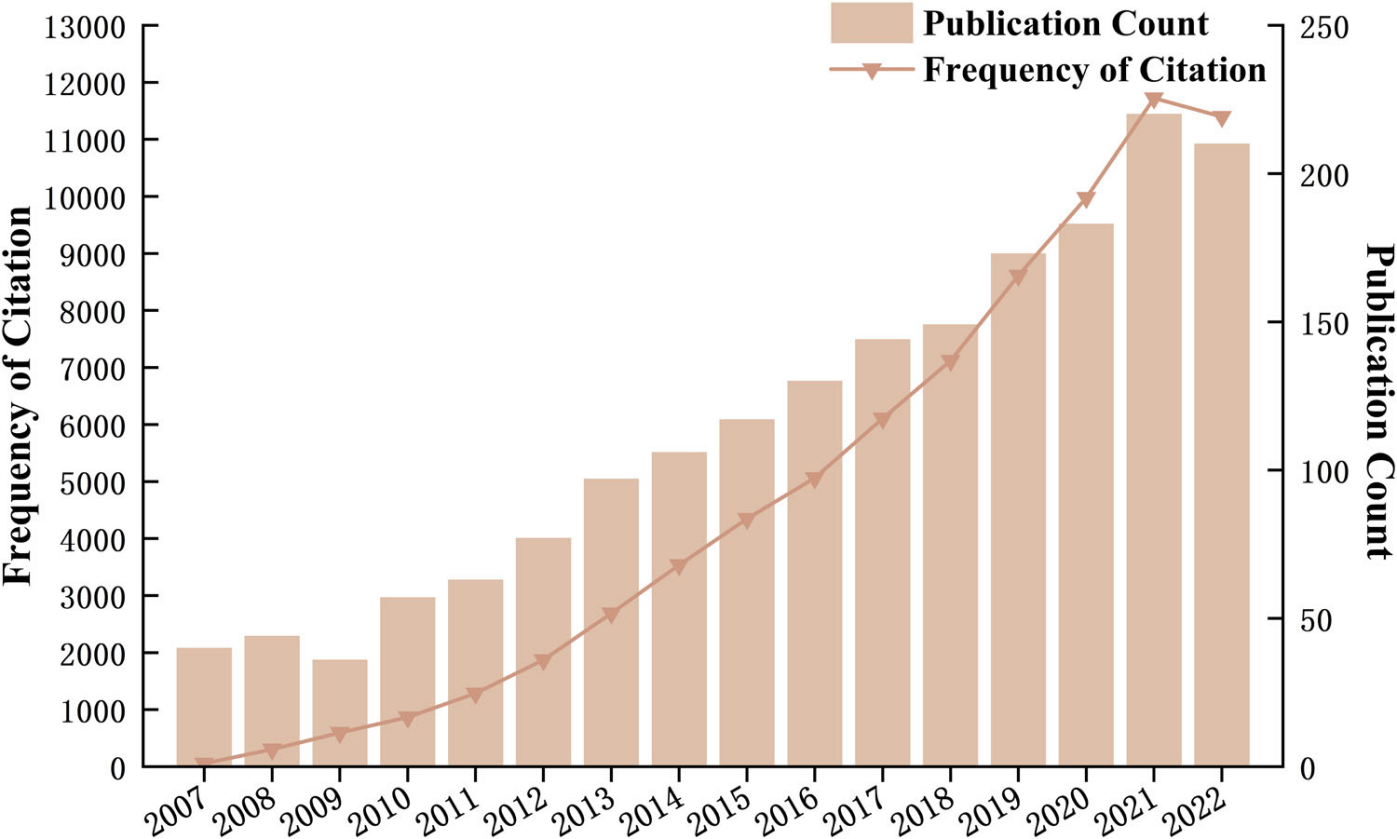
Global trend and continuous growth rate of annual publications related to neural circuits from 2007 to 2022. [Color figure can be viewed at wileyonlinelibrary.com]

We searched 1845 eligible publications in the WOSCC database over the past 15 years (2007–2022) and included them in the final bibliometric analysis (Figure 2). Through the citation report of the WOSCC database, we found that there were 76,759 citations in total, 74,963 citations excluding self‐citations, and 41.6 citations per article. Within 15 years, the number of publications on neural circuit research has shown an overall upward trend. It is worth noting that 2009–2010 had the largest time growth rate of publications in the past 15 years, which was 58%. However, 2020–2021 (183–220 articles published annually) emerged as the period with the largest increase in the number of publications in the past 15 years, and the number of publications has increased by 37 at the same time. At the same time, 2020–2021 (9976–11723 citations per year) presented the fastest‐growing period in 15 years, with an increase of 1747 citations. This also indicates that more and more attention is being paid to research in the field of neural circuits.

### 3.2 Country/region contribution analysis

**Figure 3:**
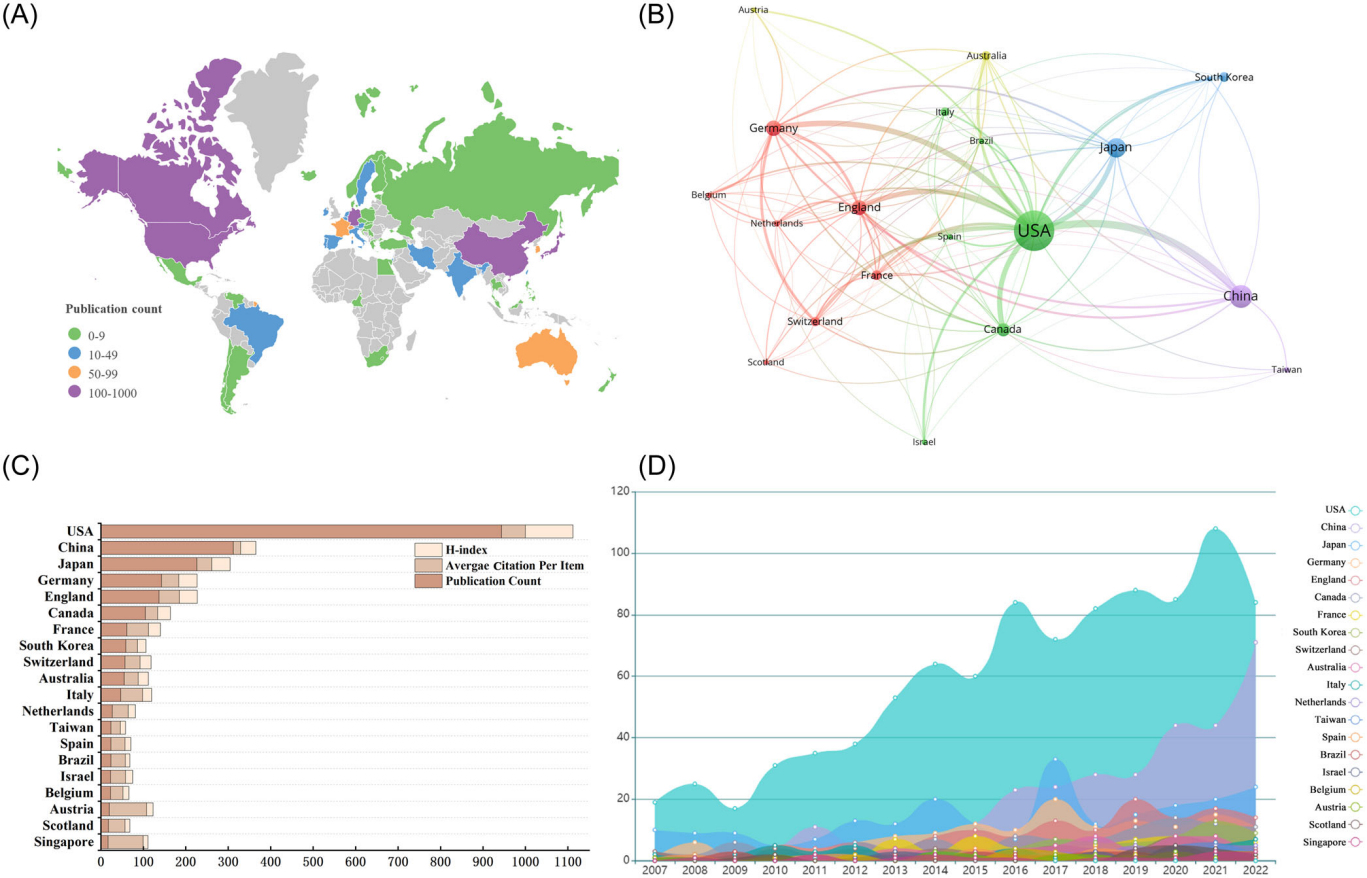
Contribution of countries/regions to the research field of neural circuits. (A) A world map of the global distribution in the field of neural loop research. According to the number of published articles, different countries are represented by different colors. (B) The distribution and international cooperation of countries/regions involved in neural circuit research. The thickness of lines reflects the frequency of cooperation; the thicker the line, the stronger the cooperation. (C) The total number of publications, the average citation of each item and the *H* index of countries that have contributed more than 15 publications in this field. (D) The increasing trend of the number of publications in countries/regions that have contributed more than 15 publications to the study of neural circuits from 2007 to 2022. [Correction added on 1 March 2024, after first online publication: In the preceding sentence, the word “methamphetamine” was replaced with “neural circuits” in this version.] [Color figure can be viewed at wileyonlinelibrary.com]

We analyzed the contribution of each country in the field of neural circuits through VOSviewer and showed the global distribution of publications through Scimago Graphic visualization data. A total of 56 countries have participated in cooperation and contributed (Figure 3A). After that, we set the minimum number of documents issued by countries to 15, and a total of 20 countries reached the threshold (Table 1). Among them, the United States (944) published the most articles, followed by China (312) and Japan (226). From the number of citations in each article, Austria (88) has the largest number of citations, followed by Singapore (82) and the United States (55). However, in terms of *H* value, the United States (112) has the highest *H* value, followed by Germany (43) and Japan (43) (Figure 3B,C). Finally, the country with the highest overall contact strength is the United States (385), followed by Germany (156) and the United Kingdom (141) (Figure 3B). This shows that the United States has an important influence on the research of neural circuits. In addition, the United States has been leading in the publications output over the period 2007–2022, China exhibited a gradually prominent increase in the related publications from 2015, Japan presented a sharp augmentation in 2017 (Figure 3D). [Correction added on 1 March 2024, after first online publication: The preceding sentence as been revised in this version.]

**Table 1:** Top 20 countries/regions that have published research publications on neural circuits.

Country/region	Publications	Citations	Average citations rate	*H*‐index
The United States	944	52,690	55.82	112
China	312	5421	17.38	36
Japan	226	8019	35.48	43
Germany	143	5806	40.60	43
England	137	6610	48.25	42
Canada	105	3026	28.82	30
France	61	3127	51.26	28
South Korea	59	1605	27.20	20
Switzerland	57	2024	35.51	26
Australia	55	1809	32.89	24
Italy	47	2424	51.57	21
Netherlands	27	1019	37.74	17
Brazil	24	805	33.54	11
Spain	24	785	32.71	14
Taiwan	24	532	22.17	12
Israel	23	808	35.13	17
Belgium	23	664	28.87	14
Austria	20	1763	88.15	15
Scotland	18	696	38.67	12
Singapore	17	1408	82.82	11

### 3.3 Institutional distribution analysis

**Figure 4:**
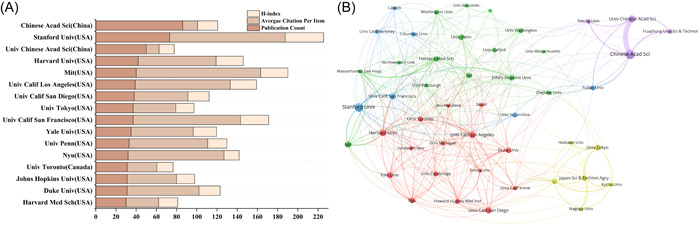
The contribution of various institutions in the research field of neural circuits. (A) The total number of publications, average citation and *H* index of institutions that have contributed at least 15 papers in this field. (B) Mapping of coauthored analysis among the 47 most productive institutions in neural loop research. Each node represents an institution, and the node size represents the number of publications. The connection between nodes indicates cooperation, and the thickness of lines indicates strength (weight on total link strength). [Color figure can be viewed at wileyonlinelibrary.com]

The top 10 institutions with the most publications are located in the United States and China (Table 2). The Chinese Academy of Sciences (86) has the largest number of publications, followed by Stanford University (73) and the University of the Chinese Academy of Sciences (50). The results also show that the publications of the Chinese Academy of Sciences are mainly concentrated in 2018, and the publications of the University of the Chinese Academy of Sciences and Harvard University are mainly concentrated in 2019 and 2015, respectively. Massachusetts Institute of Technology (MIT) (123 times), Stanford University (114 times), and the University of California, San Francisco (106 times) have the most citations per article. According to *H*, Stanford University (38) has the highest value, followed by the University of California, San Francisco (28), and Harvard University (27) (Figure 4A). We set the minimum number of articles issued by institutions to 15, and 47 cooperative institutions reached the threshold. The total contact strength of the Chinese Academy of Sciences (76) was the highest, followed by the University of the Chinese Academy of Sciences (65) and Harvard University (43) (Figure 4B). A close academic connection was found between the Chinese Academy of Sciences and the University of the Chinese Academy of Sciences, but the international connection is almost absent between China and the United States.

**Table 2:** The top 20 institutions with the most publications in the field of neural circuit research.

Institutions	Publications	Citations	Average citation rate	Total link strength
Chinese Acad Sci	86	1263	14.69	76
Stanford Univ	73	8364	114.58	36
Univ Chinese Acad Sci	50	630	12.60	65
Harvard Univ	42	3236	77.05	43
MIT	40	4927	123.18	32
Univ Calif Los Angeles	39	3669	94.08	41
Univ Calif San Diego	38	2020	53.16	23
Univ Calif San Francisco	37	3929	106.19	27
Univ Tokyo	37	1561	42.19	17
Yale Univ	35	2150	61.43	24

### 3.4 Analysis of journal distribution

**Figure 5:**
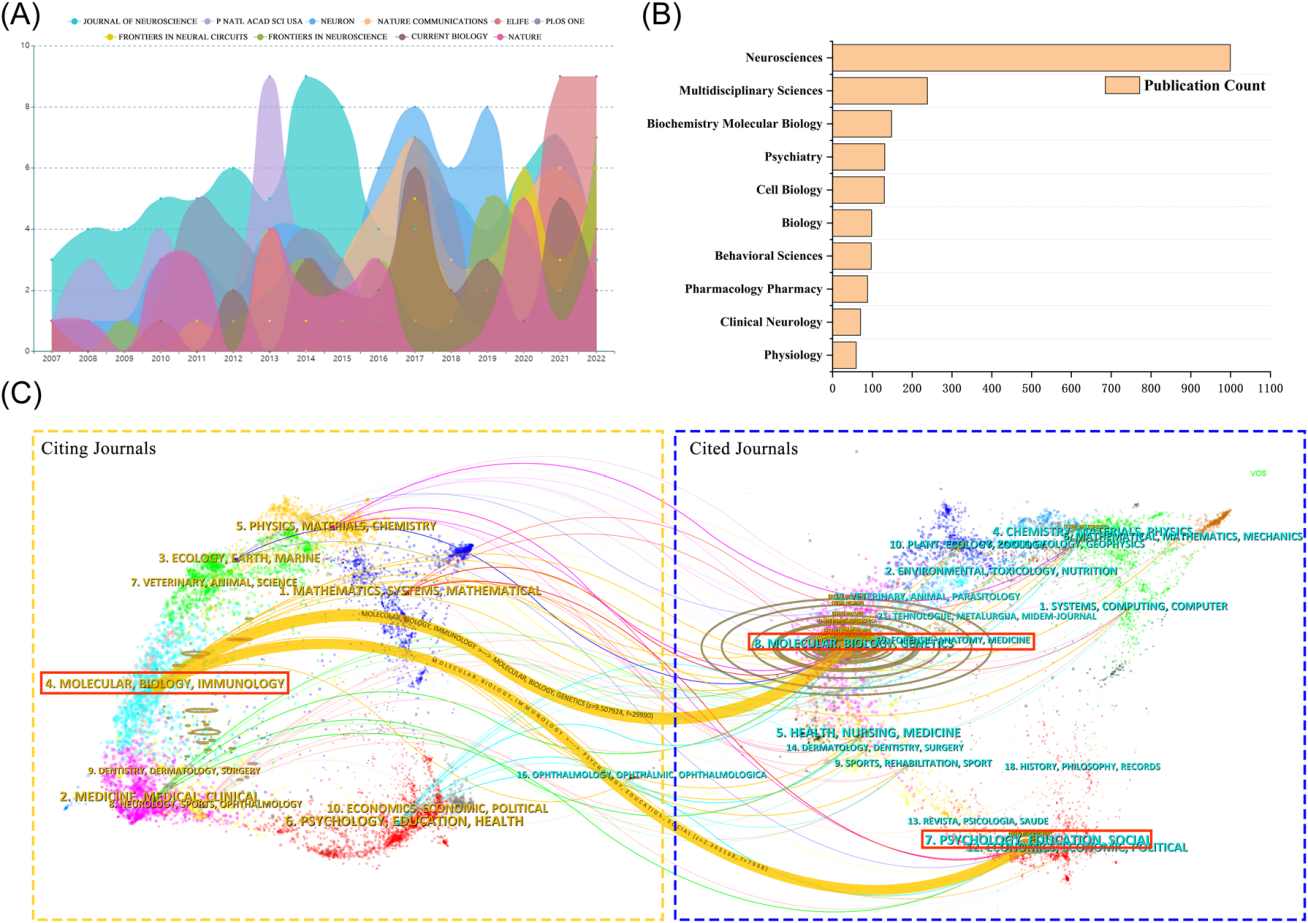
Contribution of journals in the field of neural circuit research. (A) From 2007 to 2022, the publishing volume of the top 10 productive journals of neural loop research increased. (B) The top 10 journal types with the largest number of publications on neural circuit research. (C) Double‐map superposition of journals on methamphetamine research generated by Citespace. These labels represent different research topics covered by journals. The citing journals are on the left, and the other side of the map represents the cited journals. Lines with different colors correspond to different paths of reference, starting from the reference map and ending at the reference map. The main cited journals and cited journals are displayed in the red box. [Color figure can be viewed at wileyonlinelibrary.com]

The analysis of journal distribution is helpful in understanding the popular journals in the field of neural circuits. In general, 466 journals published research related to neural circuits from 2007 to 2022. The impact index of the top 10 journals with the most published papers is shown in Table 3, most of which are classified as Q1 (70%, 7/10). Among the top 10 journals, JOURNAL OF NEUROSCIENCE has the largest number of published articles, with a total of 82. In 2014, the number of published articles in this journal reached 9. However, from 2021 to 2022, ELIFE published the most, with a total of 18 articles published within 2 years (Figure 5A). Figure 5B shows the top 10 journal types with the largest number of neural circuit research publications, of which “Neuroscience” is far ahead of other journal types. Then, the relationship between the top 10 articles cited and journals was discussed. Table 4 shows the top 10 references cited the most, most of which are from NATURE REVIEWS NEUROSCIENCE (33.3%, 3/10). Figure 5C shows the citations and citation coverage of periodicals, primarily including two categories of citation: (1) MOLECULAR, BIOLOGY, IMMUNOLOGY (the cited periodicals) and MOLECULAR, BIOLOGY, GENETICS (the cited periodicals); (2) MOLECULAR, BIOLOGY, IMMUNOLOGY (the quoted periodicals) and PSYCHOLOGY, EDUCATION, SOCIAL (the quoted periodicals).

**Table 3:** The influence index of the top 10 journals with the largest number of articles related to neural circuits.

Journal	Publication count	Impact factor (2022)	Journal citation reports	Total citations	Average citation per item
JOURNAL OF NEUROSCIENCE	82	5.3	Q1	3038	37.05
PROCEEDINGS OF THE NATIONAL ACADEMY OF SCIENCES OF THE UNITED STATES OF AMERICA	60	11.1	Q1	3324	55.4
NEURON	59	15.76	Q1	6975	118.22
NATURE COMMUNICATIONS	38	16.6	Q1	1592	41.89
ELIFE	35	7.7	Q1	737	21.06
PLOS ONE	34	3.7	Q2	896	26.35
FRONTIERS IN NEURAL CIRCUITS	33	3.5	Q3	796	24.12
FRONTIERS IN NEUROSCIENCE	31	4.3	Q2	661	21.32
CURRENT BIOLOGY	30	9.2	Q1	1072	35.73
NATURE	30	64.8	Q1	6685	222.83

**Table 4:** Top 10 most cited references in the field of neural circuits.

Title	Corresponding author	Journal	IF	Publication year	Total citations	Research direction
Neurotrophin regulation of neural circuit development and function	Poo, Mu‐ming	NATURE REVIEWS NEUROSCIENCE	38.755	2013	1234	Neurosciences & Neurology
Microbiota‐generated metabolites promote metabolic benefits via gut‐brain neural circuits	Mithieux, Gilles	CELL	66.850	2014	1218	Biochemistry & Molecular Biology, Cell Biology
A resource of Cre driver lines for genetic targeting of GABAergic neurons in cerebral cortex	Huang, Z. Josh	NEURON	18.688	2011	1120	Neurosciences & Neurology
Increasing adult hippocampal neurogenesis is sufficient to improve pattern separation	Sahay, Amar	NATURE	69.504	2011	1048	Science & Technology—Other Topics
The contextual brain: implications for fear conditioning, extinction, and psychopathology	Maren, Stephen	NATURE REVIEWS NEUROSCIENCE	38.755	2013	938	Neurosciences & Neurology
The neural circuit of orexin (hypocretin): maintaining sleep and wakefulness	Sakurai, Takeshi	NATURE REVIEWS NEUROSCIENCE	38.755	2007	868	Neurosciences & Neurology
How does the brain solve visual object recognition?	DiCarlo, James J.	NEURON	18.688	2012	854	Neurosciences & Neurology
ApoE‐directed therapeutics rapidly clear beta‐amyloid and reverse deficits in AD mouse models	Landreth, Gary E.	SCIENCE	63.832	2012	808	Science & Technology—Other Topics
A toolbox of Cre‐dependent optogenetic transgenic mice for light‐induced activation and silencing	Zeng, Hongkui	NATURE NEUROSCIENCE	28.771	2012	799	Neurosciences & Neurology
Rapid regulation of depression‐related behaviors by control of midbrain dopamine neurons	Han, Ming‐Hu	NATURE	69.504	2013	715	Science & Technology—Other Topics

### 3.5 Author distribution analysis

**Figure 6:**
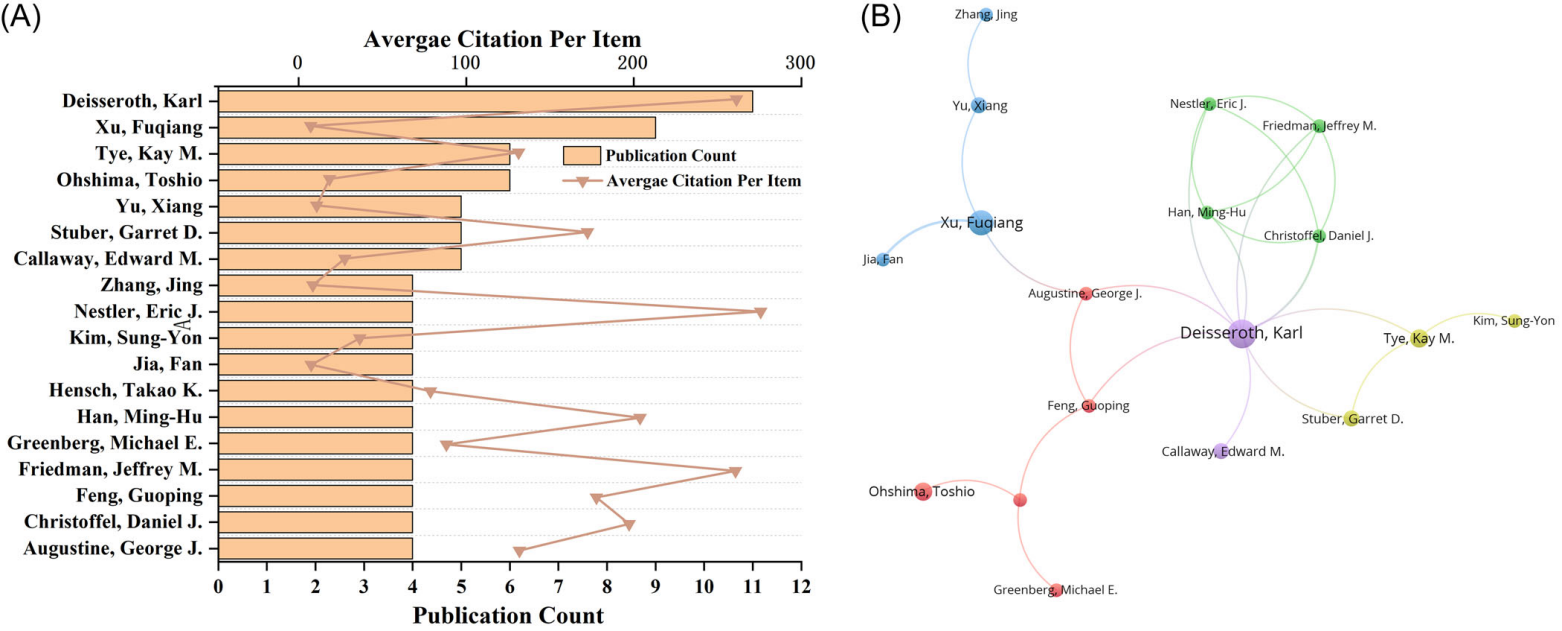
The author's contribution in the field of neural circuit research. (A) The total number of publications of authors who have contributed at least seven articles in this field and the average of each citation. (B) According to VOSviewer, the mapping of coauthored analysis among the authors who have published at least 4 papers on neural loop research. Each node represents an author, and the node size represents the number of publications. [Correction added on 1 March 2024, after first online publication: In the preceding sentence, the word “institution” was replaced with “author” in this version.] The connection between nodes indicates cooperation, and the thickness of lines indicates strength (weight on total link strength). [Color figure can be viewed at wileyonlinelibrary.com]

The most prolific authors with the largest number of publications and citations from 2007 to 2022 were listed in Table 5. The number of articles citations is an important indicator for measuring the influence of authors. The results show that the authors with the largest number of publications are mainly from the United States and China, while the authors with the largest number of citations are mainly from the United States and France. In the United States, Karl Deisseroth of New York University has published the most articles in the field of neural circuits. They have participated in publishing 11 articles in total, while Karl Deisseroth owned the highest number of citations, totaling 2874 times. Xu Fuqiang, from the Chinese Academy of Sciences, published the most articles in China, making contributions in publishing nine articles. Although Nestler and Eric J. of the United States only published four articles in this field, they have 275 times of citations for each article, followed by Karl Deisseroth, whose article was cited 261 times each (Figure 6A), indicating that these two authors have great influence in this field. We set the minimum number of cooperative articles to 4, and a total of 18 authors reached the threshold. Karl Deisseroth (10) had the highest total contact strengths (Figure 6B). Therefore, Karl Deisseroth is the most influential author in the field of neural circuit research.

**Table 5:** Top authors in the field of neural circuit research are ranked according to the number of publications and citations.

Author	Country	Affiliation	Publications	Citations
Deisseroth, Karl	the United States	Stanford University	11	2874
Wang, Xiao‐Jing	the United States	Yale School of Medicine	11	641
Luo, Liqun	the United States	Stanford University	10	959
Xu, Fuqiang	China	Chinese Acad Sci	9	65
Gong, Hui	China	Huazhong Univ Sci & Technol	8	212
Eickhoff, Simon B.	Germany	Res Ctr Julich	7	441
Kalin, Ned H.	the United States	University of Wisconsin Madison	7	396
Li, Anan	China	Huazhong Univ Sci & Technol	7	179
Deisseroth, Karl	the United States	Stanford University	11	2874
Barres, Ben A.	the United States	Stanford University	4	2352
Zeng, Hongkui	the United States	Allen Institute for Brain Science	4	1391
Mithieux, Gilles	France	UDICE‐French Research Universities	4	1317
De Vadder, Filipe	France	CNRS ‐ National Institute for Biology (INSB)	2	1278
Duchampt, Adeline	France	Universite Claude Bernard Lyon 1	2	1278
Goncalves, Daisy	France	UDICE‐French Research Universities	2	1278
Khakh, Baljit S.	the United States	University of California Los Angeles	3	1268
Huang, Z. Josh	the United States	Duke University	3	1229
Taniguchi, Hiroki	Japan	Nagoya University	2	1201

### 3.6 Keyword co‐occurrence cluster analysis

**Figure 7:**
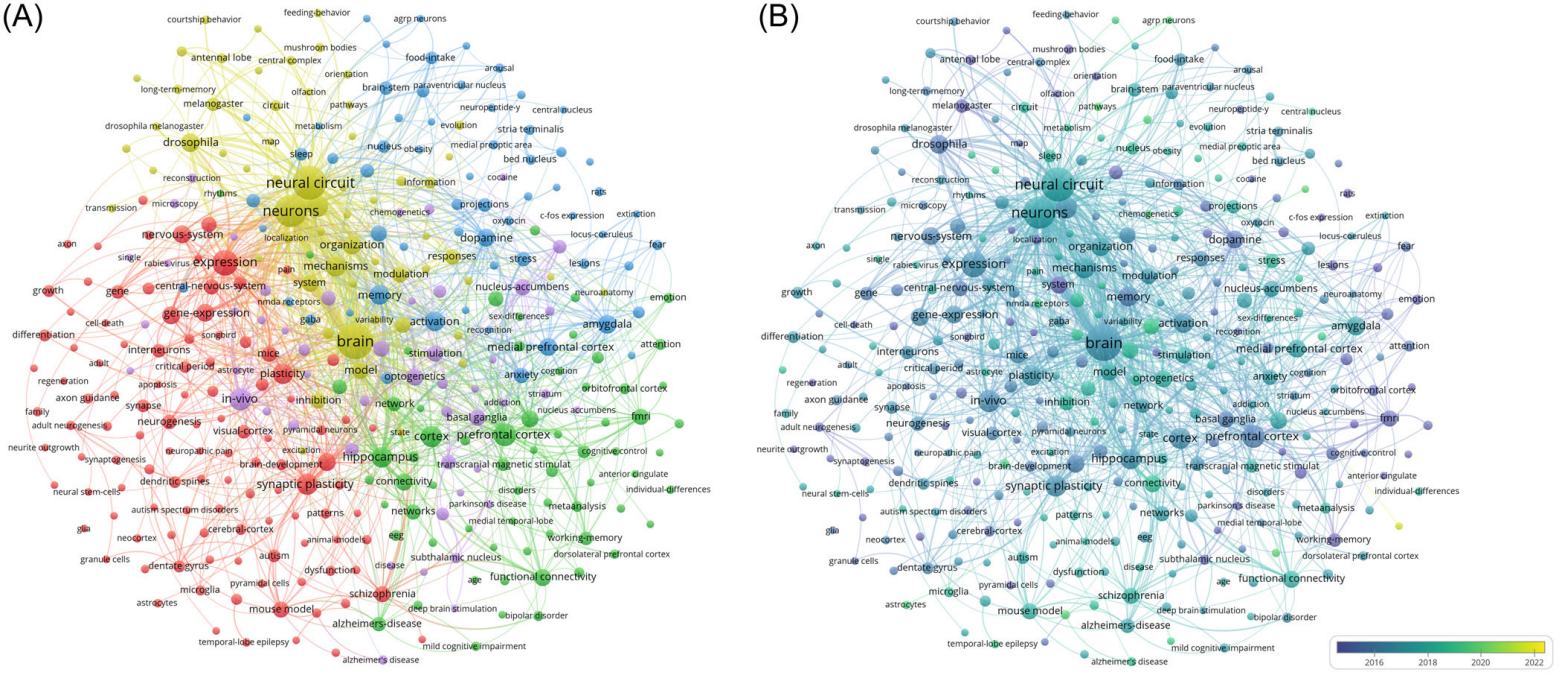
The contribution of keywords in the field of neural loop research. (A) VOSviewer conducted keyword co‐occurrence analysis on neural circuit research. Different colors indicate keywords in certain category. (B) Superimposed visualization of keyword co‐occurrence analysis. Purple nodes represent earlier keywords, while yellow nodes reflect recent keywords. [Color figure can be viewed at wileyonlinelibrary.com]

The VOSviewer is used to analyze keywords. The threshold value is set to at least 10 keyword occurrences. A total of 348 keywords were identified (Figure 7A). To observe whether keywords change over time, we display them in different colors in an overlay visualization and display their annual distribution according to the average release year (Figure 7B). Keywords such as “core,” “basal ganglia,” “basolateral amygdala,” and “fMRI” mainly appeared in 2015. In recent years, keywords such as “dynamics,” “circuits,” “functional connectivity,” “optogenetics,” “amyloid beta,” and “major repressive disorder” have appeared. The change of keywords from 2015 to recent years shows the evolution from a single brain region to neural circuit exploration. The keywords emerging in recent years indicate that the neural circuit mechanism is increasingly popular for the treatment of diseases. Table 6 lists the top 20 keywords with the highest frequency from 2007 to 2022. By comprehensive keyword analysis, it can be found that the connection of neural circuits in vivo and the performance of behavioral mechanisms may be the research hotspot in recent years, and it shows the future research direction.

**Table 6:** The top 20 keywords with the highest frequency in neural loop research.

Keyword	Occurrence	Total link strength	Average publication year
Brain	321	2219	2016.69
Neural circuit	265	1901	2017.51
Neurons	248	1630	2017.40
Expression	141	958	2016.65
Prefrontal cortex	114	870	2015.96
In‐vivo	102	700	2016.38
Cortex	96	702	2016.64
Behavior	96	678	2016.55
Synaptic plasticity	94	689	2016.55
Hippocampus	92	760	2016.55
Mechanisms	87	578	2017.08
Plasticity	86	623	2016.86
Memory	83	622	2015.96
Activation	81	596	2016.65
Organization	74	541	2017.22
*Drosophila*	72	535	2015.79
Amygdala	71	545	2017.30
Gene‐expression	66	436	2016.53
Medial prefrontal cortex	65	526	2017.51
Nervous‐system	65	395	2016.08

## 4 DISCUSSION

The nerve circuit refers to the circulation link between interconnected neuronal groups in the brain, which is one of the basic mechanisms for controlling behavior and cognition.^25^, ^26^ In recent years, more and more attention has been paid to the study of neural circuits, and bibliometric analysis has become one of the hot spots in the research field. Therefore, to analyze the global trend of neural circuits and predict the future research direction, this study conducted a comprehensive interpretation of 1845 publications on neural circuits included in the WOS database from 2007 to 2022, from the perspective of publications output, countries, institutions, journals, authors, and keywords. Our results demonstrated the research on neural circuits showed a growing trend in this period, especially in the past decade, when the number of relevant publications grew sharply in many countries like the United States, China, Germany, the United Kingdom, Japan, and so forth, reflecting the increasing influence of neural circuit research on the academic community. In the national and regional analyses, the United States ranked first in terms of publications output and the number of authors investigating this field, followed by China. However, the United States stood out in this field with more highly influential researchers, investigations, and citations than China. When looking into the research institutions, 80% of the top 10 institutions are located in the United States, in which Stanford University has the largest number of articles, and MIT has the largest number of citations, while in China, the largest number of publications comes from the Chinese Academy of Science. From the perspective of institutional contact, little institutional contact was observed among the Chinese Academy of Science, Stanford University and Harvard University, despite close connection existing among institutions within own countries, for instance, close links found between University of Chinese Academy of Sciences and Chinese Academy of Sciences. Therefore, active cooperation between the institutions of the two countries requires to be strengthened, which may lead to the substantial development of global neural circuit research.

In general, 466 journals published articles related to neural circuits from 2007 to 2022. We screened the top ten journals publishing the most articles, 70% of which were classified as JCR Q1. Among them, JOURNAL OF NEUROSCIENCE has published the most papers (82) and NEURON has the most citations (6975 times). Although NATURE has the least number of documents, it has the highest citation rate (222.83). JOURNAL OF NEUROSCIENCE is one of the important journals in the field of neuroscience, published by the Society for Neuroscience in the United States with a wide international readership. Therefore, articles accepted for publication by this journal are usually considered to have a high academic reputation. Nevertheless, we can see that in the academic community, researchers typically tend to cite articles from highly influential journals, which are often considered to have higher credibility and academic reputation. Therefore, investigators should also take into account factors such as domain‐specific journals, research quality, and academic influence when citing the literature. The most important thing is that citations should be based on scientific content and reliability rather than just the impact factors of the journal.

As far as the distribution of authors is concerned, we have screened the authors based on the number of articles ≥7 and the number of citations ≥1200, respectively. Among them, Karl Deisseroth (Stanford University) from the United States ranks first in terms of the number of articles, citations, and contact strength and is the most productive author. Dr. Deisseroth received his medical degree and a doctorate in neuroscience from Stanford University School of Medicine. During his training, he developed a keen interest in uncovering the underlying mechanisms of brain disorders and finding innovative approaches to their treatment. Karl Deisseroth is a renowned neuroscientist from the United States who first proposed optogenetics in 2006, a revolutionary technique that combines optics and genetics to control specific neurons with light‐sensitive proteins, making significant contributions to the field of optogenetics and the study of neural circuits. His groundbreaking research on understanding the intricate workings of the brain and neural circuits allowed researchers to manipulate and study neural activity with unprecedented precision, offering new insights into how neural circuits function and how they are implicated in various neurological (AD and PD) and psychiatric disorders diseases such as depression, anxiety, addiction, and schizophrenia.^27^, ^28^, ^29^, ^30^ There are three institutions in China with a total number of related publications ≥7, mainly from Huazhong University of Science and Technology. However, most of the authors with more than 1200 citations came from the United States and France. Therefore, China's research on neural circuits should be based on the advancements of Dr. Deisseroth in understanding the complexities of the brain and functions of neural circuits for the genuine development of targeted therapies for neurological and psychiatric disorders.

Keyword clustering analysis can not only clearly show the evolution of a research field but also reflect the changing direction of research hot spots. In this study, we uncovered the evolution of some keywords with a large number of occurrences, focusing more on “neural circuits,” “functional connectivity,” “optogenetics,” “dynamics,” “neural circuit disruptions,” and so on. The changes in keyword searching revealed the reformation of detection technology in brain research and the heated research focus of neuroscience on neural circuits. Optogenetics has several advantages when compared to functional magnetic resonance imaging (fMRI). Through genetic manipulation and light‐sensitive proteins, optogenetics allows for selective activation or inhibition of specific types or regions of neurons, providing insight into the function and interactions of distinct neuronal populations.^31^ Moreover, optogenetics offers spatial localization at the cellular level, enabling researchers to directly control or record the activity of specific neuronal populations. However, fMRI provides information about overall brain region activity with relatively low spatial resolution. Optogenetics can achieve very high temporal resolution, capturing rapid changes in neural activity, even at the sub‐second level, while fMRI typically operates on a timescale of several seconds. The research on neural circuit structure mainly focuses on the connection mode and topological structure of neurons in the brain, including brain circuits, neural networks, and so forth. Therefore, the development of optogenetics has certainly facilitated the development of research on neural circuits and revolutionized the brain science field.^32^ Studying neural circuit function enhances understanding of the role of neural circuits in various cognitive, emotional, and behavioral processes, including decision‐making, learning, and attention regulation.^14^, ^33^, ^34^ Neural circuits‐based findings have made great contributions to investigating the pathological mechanisms of neurological diseases, such as AD, Parkinson's disease, and schizophrenia.^20^, ^35^, ^36^, ^37^ Neural circuit reconstruction therapy presents as a novel therapeutic approach that has shown promising outcomes in treating various nervous system disorders and has emerged as a rapidly developing field. While the efficacy of this treatment method requires further investigation and validation, it offers a new perspective and method for treating patients, giving them renewed hope.

## 5 CONCLUSIONS

Summarily, this study, for the first time, comprehensively summarizes and analyzes the global research overview of neural circuit research. In the past 15 years, the number of high‐quality publications in this field has increased significantly. The United States maintained a top position worldwide, making the most significant contributions in the field of neural circuits research. Though significant advancements have been made in the study of neural circuits, ongoing research should take active cooperation across different countries and refer to highly influential advancements into necessary consideration to further explore the intricate network of interconnections in the brain.

## AUTHOR CONTRIBUTIONS

Zhong‐Fu Zuo is responsible for research design and manuscript revision. Ruo‐Lan Du and David H. Mauki are responsible for data collection, analysis, picture production, and manuscript drafts. All the authors strictly reviewed and approved the final manuscript.

## CONFLICT OF INTEREST STATEMENT

The authors declare no conflict of interest.

## ETHICS STATEMENT

Not applicable.

## Data Availability

The data analyzed during the current study are available from the corresponding author upon reasonable request.
